# African ancestry-derived *APOL1* risk genotypes show proximal epigenetic associations

**DOI:** 10.1186/s12864-024-10226-0

**Published:** 2024-05-08

**Authors:** Charles E. Breeze, Bridget M. Lin, Cheryl A. Winkler, Nora Franceschini

**Affiliations:** 1 Division of Cancer Epidemiology and Genetics, National Cancer Institute, National Institutes of Health, Bethesda, MD USA; 2 Department of Biostatistics, University of North Carolina, Chapel Hill, NC USA; 3 Cancer Innovation Laboratory, National Cancer Institute, National Institutes of Health, Basic Research Program, Frederick National Laboratory, Frederick, MD USA; 4 Department of Epidemiology, University of North Carolina, Chapel Hill, NC USA

**Keywords:** DNA methylation, APOL1 risk variants, chronic kidney disease, methylation quantitative trait locus (meQTL), epigenetic, gene regulation

## Abstract

Apolipoprotein L1 (*APOL1*) coding variants, termed G1 and G2, are established genetic risk factors for a growing spectrum of diseases, including kidney disease, in individuals of African ancestry. Evidence suggests that the risk variants, which show a recessive mode of inheritance, lead to toxic gain-of-function changes of the APOL1 protein. Disease occurrence and presentation vary, likely due to modifiers or second hits. To understand the role of the epigenetic landscape in relation to *APOL1* risk variants, we performed methylation quantitative trait locus (meQTL) analysis to identify differentially methylated CpGs influenced by *APOL1* risk variants in 611 African American individuals. We identified five CpGs that were significantly associated with *APOL1* risk alleles in discovery and replication studies, and one CpG-*APOL1* association was independent of other genomic variants. Our study highlights proximal DNA methylation alterations that may help explain the variable disease risk and clinical manifestation of *APOL1* variants.

**Supplementary Information:**

The online version contains supplementary material available at 10.1186/s12864-024-10226-0.

## Main text

Two *APOL1* risk variants (G1 and G2) have been identified as genetic risk factors for a wide spectrum of diseases including chronic kidney disease (CKD), hypertension-attributed kidney failure (odds ratio [OR] 7), HIV-1 associated nephropathy (OR 29–89), focal segmental glomerulosclerosis (FSGS) (OR 17), and more recently, COVID-19-associated nephropathy and pregnancy-induced hypertension (preeclampsia) in individuals of African descent [1, 2]. Our studies in African American postmenopausal women additionally identified associations of *APOL1* risk variants with heart failure [3]. These diseases contribute to substantial morbidity and mortality as well as poor maternal and fetal outcomes. *APOL1* is a gene involved in innate immunity, and G1/G2 variants are believed to be under recent selective pressure driven by resistance to African trypanosomiasis [1]. The prevalence of *APOL1* high risk genotypes comprising any two risk alleles is 13% in African American individuals and ~ 1% in Hispanics/Latinos of African descent [4, 5]. The mechanisms relating *APOL1* to disease and possible treatment are under investigation [4, 6]. Some new therapies in this area are based on the hypothesis that *APOL1* G1/G2 variants are related to gain-of-function protein toxicity [7] and exploit the observation that the APOL1 protein is not required for life since middle-aged humans carrying two null alleles are healthy, and most non-human primates and all non-primate mammals lack the *APOL1* gene [8, 9].

The relationship between *APOL1* risk variants and the epigenome has been understudied and could provide insights into targets to prevent or treat *APOL1* associated diseases. Differences in DNA methylation (DNAm) can be driven by genomic sequence variants and can modulate local gene expression. Studies have shown that many functionally relevant changes in DNAm occur in regulatory elements such as enhancers [10]. Prior studies identified African ancestry-related differential gene expression of *APOL1* in hepatocytes of African American individuals [11]. To understand the relationship between *APOL1* risk variants and the epigenome, we performed DNA methylation quantitative trait locus (meQTL) analysis in African Americans. The number of G1 and G2 risk alleles were combined for analyses (see Methods).

Whole blood DNAm was measured using the Illumina 450 K array in two studies sampled from the Women’s Health Initiative (WHI), a cohort study of postmenopausal women aged 50–79 years. Analysis was restricted to African American participants (WHI-BAA23, *n* = 410 discovery; WHI-EMPC, *n* = 201 replication) who also had *APOL1* risk genotypes. *APOL1* G1 and G2 risk were computed as 0, 1 or 2 copies of the risk alleles. We used linear models to test the association of DNA methylation with *APOL1* allele copies in models adjusted for age, recruitment center, smoking status (current, past, never) and smoking pack-years, blood cell composition, batch effects and the first 10 principal components derived from genome-wide genotypes. For each associated CpG we compared the consistency of findings between cohorts (WHI-BAA23 and WHI-EMPC studies).

**Fig. 1:**
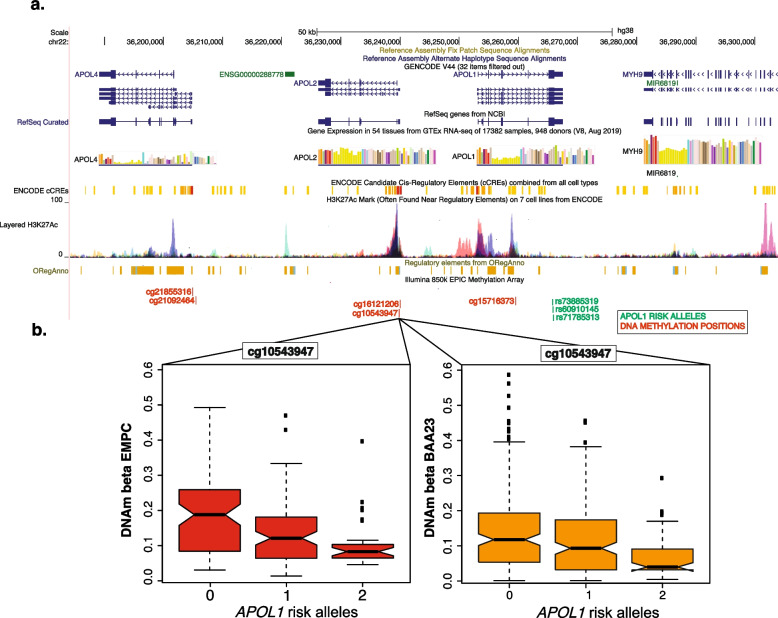
The proximal epigenetic landscape of *APOL1* risk alleles: **A** Shown is the chromosome region with genes (top), and histone marks (middle) from Encyclopedia of DNA Elements (ENCODE) data. **B** Shown are meQTL CpGs for *APOL1* G1/G2 number of risk alleles (0, 1 or 2). An example of a DNAm position associated with *APOL1* genotypes is highlighted at cg10543947 showing consistent direction of effect in two distinct studies (WHI-EMPC and WHI-BAA23). Replicated significant CpGs are shown in red

The average age of participants was 61.8 and 61.0 years and the average estimated glomerular filtration rate (eGFR) was 92.3 and 93.6 ml/min/1.73m^2^ for participants of WHI-BAA23 and WHI-EMPC, respectively. Chronic kidney disease (defined by an eGFR< 60 ml/min/1.73m^2^) was present in 6% of participants. Two copies of *APOL1* risk alleles were present in 13% of participants (Table S1). We identified five CpGs that were significantly associated with the number of *APOL1* risk alleles after Bonferroni correction, all of which replicated, showing consistent association and direction of effect (Table S2, Fig. S1). Figure 1 shows the location of identified meQTL CpGs and an example at cg10543947, which is in a candidate regulatory region (Fig. 1a) and, like other significant meQTL CpGs, shows consistent direction of effect in both cohorts (Fig. 1b**,** Table S2).

Table S3 shows additional single nucleotide polymorphisms (SNPs) that were significantly associated with four out of the five CpGs in models adjusting for the number of *APOL1* risk alleles. Specifically, we identified additional significant meQTLs for 4 of the 5 CpGs (cg10543947, cg15716373, cg16121206, and cg21855316). The additional identified meQTLs for these 4 CpGs were also shown to attenuate *APOL1* association with DNA methylation (Table S3). For cg21092464 no additional significant meQTLs were identified, even after rerunning analysis on SNPs within 5 kb of this CpG. In short, 4 out of the 5 CpGs show association with SNPs that were also found to attenuate *APOL1* association with DNA methylation in these CpGs, while 1 out of the 5 CpGs did not. eFORGE analyses shows that 3 out of the 5 CpGs (cg10543947, cg15716373, and cg16121206) are in DNase I hotspots in kidney and other tissues, while CpG cg21855316 is in a blood enhancer, and cg21092464 is in a B cell and liver enhancer (Tables S4, S5 and S6). Taken together, these results indicate an meQTL effect of *APOL1* risk alleles that is independent of additional variants in the region, and put *APOL1* associations in context with additional genotype data.

There is little understanding of why only a subset of carriers of two *APOL1* risk variants develop disease. Prior studies suggest that *APOL1* G1 and G2 are gain-of-function variants [4]. In our study, we identify epigenetic differences at this locus, which may occur prior to chronic kidney disease, given that our participants had an average normal eGFR. Epigenetic regulation may contribute to the heterogeneity in disease risk and manifestation among individuals of African descent who carry *APOL1* risk alleles. The relationship between these epigenetic differences and gene regulation will need to be further explored, as well as their potential contribution to kidney toxicity and hypertensive conditions.

Our findings highlight epigenetic differences associated with disease risk variants, including for variants derived from African ancestry that are common in African Americans. This is an important consideration for studies focusing on disease-associated ancestry-specific variants [12, 13], given that epigenetic marks such as DNAm may be targets to modify disease risk. Novel therapies tested for APOL1-associated kidney disease include small molecule compounds that bind to the APOL1 protein to inhibit APOL1 channel function [14]. Antisense oligonucleotides that block *APOL1* transcription in *Apol1*-transgenic mice models have been shown in two independent studies to ameliorate proteinuria and reduce kidney dysfunction [15, 16]. However, at least 6 other mechanisms for *APOL1* related disease have been proposed including mitochondrial and endolysosomal dysfunction, and inflammasome pathways, which may provide targets for therapeutic intervention [17]. Our study supports research to better understand differences in the epigenetic landscape associated with *APOL1* risk variants in individuals of African descent.

## Methods

### Population

WHI is a study of postmenopausal women (aged 50–79 years), comprising 161,808 women recruited from 40 U.S. clinical centers to participate in an observational study or in clinical trials during 1993–1998 [18–21]. The study has comprehensive information on risk factors including lifestyle, medical history, medication, physical measures and biomarkers obtained at a baseline clinical examination and follow-up. All participants have provided informed consent for genetic research. Two DNA methylation studies of WHI African American participants were included.

### DNA methylation profiling

Blood DNAm was assayed using the Illumina 450 K array in 3927 WHI participants from two studies: the Broad Agency Award 23 (WHI-BAA23), a case-control study of cardiovascular disease, and the Epigenetic Mechanisms of PM-Mediated Cardiovascular Disease Risk (WHI-EMPC), a stratified, random sample of participants examined between 1993 and 2001. To correct the beta value distributions of the two types of probes on the 450 K array, *β*-values were normalized using the beta-mixture quantile (BMIQ) normalization method, [22] extreme outliers were removed, and ComBat was used to adjust for technical artifacts across batches [23]. Cell proportions were estimated using the Houseman method [24].

### *APOL1* and genome-wide genotyping

*APOL1* G1 (rs73885319 & rs60910145, two amino acid substitutions: S342G and I384M) and G2 (rs71785313, two-amino acid deletion: del388N389Y) (Table S2) were directly genotyped using the Taqman assay (Thermo Fisher Scientific). *APOL1* G1 and G2 variants were coded as 0, 1 or 2 copies of the risk alleles. Genome-wide genotypes were available in approximately 8500 WHI African American women who were genotyped using the Affymetrix 6.0 array. Genetic data was imputed using TOPMed freeze 8 data. We included SNPs available in the 1000 Genomes Project AFR data, and trimmed SNPs based on linkage disequilibrium (r2 = 0.8) using SNPclip from LDlink [25]. Genome-wide genotypes were used to estimate principal components among unrelated individuals using standard methods [26].

### Statistical analysis and functional annotation

We tested the association of methylation at CpGs within 2 MB of the *APOL1* risk variants using linear models adjusted for age, recruitment center, smoking status (current, past, never), pack-years, cell composition and 10 principal components derived from genome-wide genotypes, and performed robust standard error calculations via the ‘sandwich’ package [27]. Statistical significance was considered after Bonferroni correction for the number of DNA methylation CpGs tested within the region (*n* = 972 CpGs). For each identified meQTL CpG, we compared the consistency of findings between WHI-BAA23 discovery and WHI-EMPC replication studies. We provided functional annotation using the UCSC genome browser and data from the Encyclopedia of DNA Elements (ENCODE) [28], and eFORGE (https://eforge.altiusinstitute.org/) [29, 30]. We also tested for association of SNPs with the 5 CpGs using the same covariates listed above, in models adjusted for the number of *APOL1* risk alleles with a significance threshold based on the number of SNPs tested.

### Supplementary Information

**Additional file 1: Table S1.** Characteristics of WHI African American participants used for discovery and replication of meQTL CpGs. **Table S2.** Significant meQTL CpGs of *APOL1* risk variants in WHI-BAA23 and replication in WHI-EMPC. **Table 3.** CpG association findings in models including SNPs and *APOL1*. **Table S4:** eFORGE annotations for top 5 CpGs across DNase I hotspots from the Roadmap Epigenomics consortium. **Table S5:** eFORGE annotations for top 5 CpGs across HMM chromatin states from the Roadmap Epigenomics consortium. **Table S6:** eFORGE annotations for top 5 CpGs across histone mark broadPeaks from the Roadmap Epigenomics consortium. **Fig. S1.** Manhattan plot showing CpGs associated with APOL1 risk alleles in the discovery study. X-axis shows the chromosome positions and Y-axis the -log10(*p*-value) for associations. The horizontal line is the significance threshold.

## Data Availability

This research was conducted using genotype and DNA methylation data from WHI, which is publicly available through dbGap (access phs001077.v1.p1 and phs001335.v2.p3).
