# Supplementary material for: African ancestry-derived APOL1 risk genotypes show proximal epigenetic associations
Source: BMC Genomics. 2024 May 8;25:452. doi: 10.1186/s12864-024-10226-0 (PMC11077761; doi:10.1186/s12864-024-10226-0)

Supplementary material

**Table S1.** Characteristics of WHI African American participants used for discovery and replication of meQTL CpGs.

| Characteristics | WHI-BAA23 (N=410) | WHI-EMPC (N=201) |
| --- | --- | --- |
| Mean age (SD), years | 61.8 (6.4) | 61.0 (6.7) |
| Mean eGFR (SD), ml/min/1.73 m^2^ | 92.3 (19.5) | 93.6 (20.1) |
| Current smoker, N (%) | 59 (14.4) | 24 (11.9) |
| Ever smoker, N (%) | 212 (51.7) | 99 (49.3) |
| Hypertension, N (%) | 233 (54.4) | 115 (57.2) |
| Diabetes, N (%) | 86 (21.0) | 34 (16.9) |
| eGFR <60 ml/min/1.73 m^2^ | 25 (6.1) | 11 (5.5) |
| *APOL1* risk variants 1 copy | 173 (42.2) | 91 (45.3) |
| *APOL1* risk variants 2 copies | 57 (13.9) | 27 (13.4) |

N=number; SD=standard deviation

**Table S2.** Significant meQTL CpGs of *APOL1* risk variants in WHI-BAA23 and replication in WHI-EMPC

| CpG | Chr: position  (hg38) | Gene | WHI-BAA23 Beta/SE (P-values) | WHI-EMPC Beta/SE (P-values) |
| --- | --- | --- | --- | --- |
| cg15716373 | chr22: 36259497 | *APOL1* | 0.0189/0.0025 (1.549E-14) | 0.0230/0.0029 (4.7E-15) |
| cg16121206 | chr22: 36240010 | *APOL2* | -0.0378/ 0.0068 (2.5E-08) | -0.0514/0.0132 (9.9E-05) |
| cg10543947 | chr22: 36239837 | *APOL2* | -0.0335/0.0061 (4.6E-08) | -0.0394/0.0112 (4.6E-04) |
| cg21092464 | chr22: 36205477 | *APOL4* | 0.0082/0.0016  (2.4E-07) | 0.0077/0.0026 (2.6E-03) |
| cg21855316 | Chr22: 36204934 | *APOL4* | 0.0290/ 0.0071 (4.8E-05) | 0.0388/0.0119 (1.1E-03) |

The significance threshold for discovery is 5.1E-05 given 972 CpGs were tested. For comparison, *APOL1* variant positions are: rs73885319-G1, chr22:36265760; rs60910145-G1, chr22:36265988; rs71785313-G2, chr22:36265996.

Tables S3-6 are included in an excel file.

**Figure S1**. Manhattan plot showing CpG associations with *APOL1* allele risk in the discovery cohort. X-axis shows the chromosome positions (hg38) and Y-axis the -log(p-value) for associations. The horizontal line is the significance threshold.


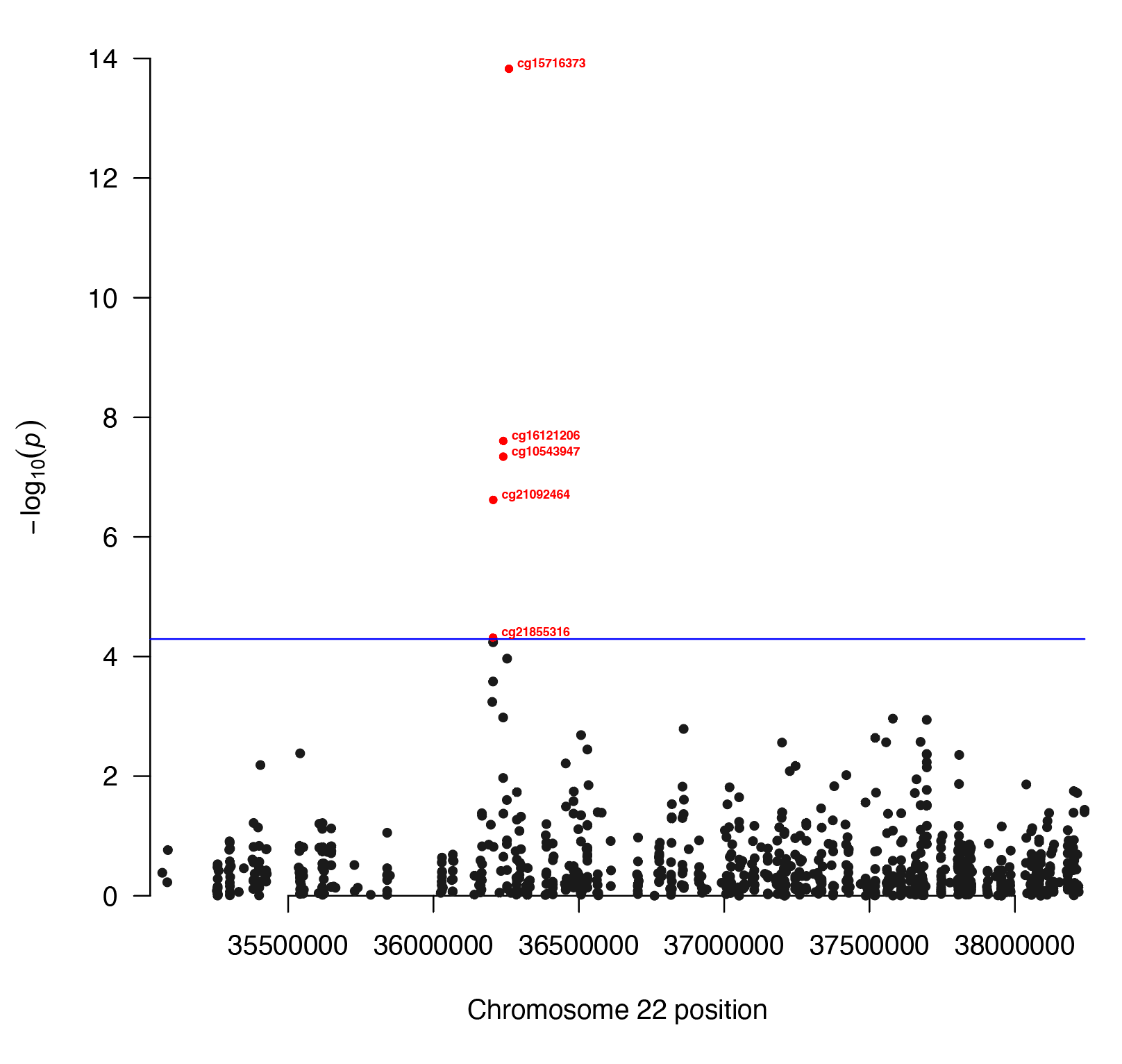

Supplement: Supplementary file 1 — Additional file 1: Table S1. Characteristics of WHI African American participants used for discovery and replication of meQTL CpGs. Table S2. Significant meQTL CpGs of APOL1 risk variants in WHI-BAA23 and replication in WHI-EMPC. Table 3. CpG association findings in models including SNPs and APOL1. Table S4: eFORGE annotations for top 5 CpGs across DNase I hotspots from the Roadmap Epigenomics consortium. Table S5: eFORGE annotations for top 5 CpGs across HMM chromatin states from the Roadmap Epigenomics consortium. Table S6: eFORGE annotations for top 5 CpGs across histone mark broadPeaks from the Roadmap Epigenomics consortium. Fig. S1. Manhattan plot showing CpGs associated with APOL1 risk alleles in the discovery study. X-axis shows the chromosome positions and Y-axis the -log10(p-value) for associations. The horizontal line is the significance threshold. [file 12864_2024_10226_MOESM1_ESM.zip › Supplementary material BMC Genom updated.28.feb.2024_ESM.docx]
